# Mechanism Sharing Between Genetic and Gestational Hypoxia-Induced Cardiac Anomalies

**DOI:** 10.3389/fcvm.2018.00100

**Published:** 2018-08-13

**Authors:** Olivia Moumne, Rajib Chowdhurry, Cassandra Doll, Natalia Pereira, Mustafa Hashimi, Tabor Grindrod, James J. Dollar, Alberto Riva, Hideko Kasahara

**Affiliations:** ^1^Department of Physiology and Functional Genomics, College of Medicine, University of Florida, Gainesville, FL, United States; ^2^Department of Pathology, Immunology and Laboratory Medicine and the Emerging Pathogens Institute, University of Florida, Gainesville, FL, United States; ^3^Bioinformatics, Interdisciplinary Center for Biotechnology Research, University of Florida, Gainesville, FL, United States

**Keywords:** cardiac anomaly, gestational hypoxia, genetic mutation, mouse models, nkx2-5

## Abstract

**Background:** Cardiac development is a dynamic process both temporally and spatially. These complex processes are often disturbed and lead to congenital cardiac anomalies that affect approximately 1% of live births. Disease-causing variants in several genetic loci lead to cardiac anomalies, with variants in transcription factor *NKX2-5* gene being one of the largest variants known. Gestational hypoxia, such as seen in high-altitude pregnancy, has been known to affect cardiac development, yet the incidence and underlying mechanisms are largely unknown.

**Methods and Results:** Normal wild-type female mice mated with heterozygous *Nkx2-5* mutant males were housed under moderate hypoxia (14% O_2_) or normoxia (20.9% O_2_) conditions from 10.5 days of gestation. Wild-type mice exposed to hypoxia demonstrate excessive trabeculation, ventricular septal defects, irregular morphology of interventricular septum as well as atrial septal abnormalities, which overlap with those seen in heterozygous *Nkx2-5* mutant mice. Genome-wide transcriptome done by RNA-seq of a 2-day hypoxic exposure on wild-type embryos revealed abnormal transcriptomes, in which approximately 60% share those from *Nkx2-5* mutants without hypoxia. Gestational hypoxia reduced the expression of Nkx2-5 proteins in more than one-half along with a reduction in phosphorylation, suggesting that abnormal Nkx2-5 function is a common mechanism shared between genetic and gestational hypoxia-induced cardiac anomalies, at least at a specific developing stage.

**Conclusion:** The results of our study provide insights into a common molecular mechanism underlying non-genetic and genetic cardiac anomalies.

## Introduction

Congenital cardiac anomalies are the most prevalent birth defects, affecting approximately 1% of live births (1–3). Despite remarkable progress in understanding cardiac development, the mechanisms underlying cardiac maldevelopment in embryos that result in malformations are largely unknown.

Cardiac development is a dynamic process both temporally and spatially. Disease-causing variants in several loci have been known to cause cardiac anomalies, and the same nucleotide variant can lead to a wide variation in the type and severity of disease (4). For instance, familial congenital cardiac malformations due to a heterozygous *NKX2-5* disease-causing variant are one of the largest sets of genetic mutations related to cardiac malformations (OMIM, NCBI), and currently nearly 40 heterozygous variants have been reported in humans (5–7). Not only in humans, but a varied range of severity of cardiac anomalies was demonstrated in *Nkx2-5* mutant mice that have a single point mutation identified in human patients, and have a nearly identical genetic background by backcrossing (8). These results suggest that non-genetic factors, influence cardiac malformations.

Considering that the majority of congenital cardiac malformations cannot be linked to specific genetic etiology, non-genetic effectors of gene regulation need to be further explored (4, 9). Epidemiological studies have indicated that gestational hypoxia, such as seen in high-altitude pregnancies, increase the risk of low intrauterine growth and low birth weight, both of which are known to increase the risk of the fetus developing cardiovascular defects. Throughout the world, about 140 million people live in high altitude environments (elevated above 2,500 meters or 8,000 ft), of whom 400,000 live in the United States (10). Oxygen concentration is decreased from 20.9% at sea level to approximately 15% at 2,500 meters (altitude chart available at https://www.higherpeak.com/altitudechart.html). Not only a high-altitude pregnancy, but also various conditions, such as maternal smoking, congestive heart failure, pulmonary diseases, acute/chronic respiratory tract infections, anemia, preeclampsia, and placental insufficiency can cause gestational hypoxia (11).

In this study, we examined gestational hypoxia-induced murine cardiac anomalies using a physiological level of hypoxia of 14% oxygen concentration, and found an interaction between non-genetic and genetic cardiac anomalies induced by the abnormal function of Nkx2-5.

## Materials and methods

### Animal models

*Nkx2-5*^+/*R*52*G*^ knock-in mice were generated as reported previously (8) and were backcrossed to 129/Sv mice purchased from Charles River Laboratories (Wilmington, MA)(129/SvPasCrl) over 10 generations. Wild-type 129/Sv female mice were bred with *Nkx2-5*^+/*R*52*G*^ males. Embryonic staging was determined by standard methods counting the morning on which the vaginal plug was found as embryonic day 0.5 (E0.5). Around noon on gestation day 10 (E10.5), pregnant female mice with weight gain (12) were placed in the hypoxic chamber (COY Lab Products, Grass Lake, MI) that was connected to nitrogen and oxygen gas. The oxygen content was gradually reduced from 20.9 to 14% over 15 min and the carbon dioxide was absorbed by Carbolime (AliMed, Inc., Dedham, MA). The cages were removed from the hypoxic chamber for approximately 10 to 15 min every day to check the mouse condition, replace bedding, water, and food, and then the cages were returned to the hypoxic chamber until approximately noon on gestation day 18 (E18.5).

On the day of delivery, newborn mice at postnatal day 1 were sacrificed and hearts were isolated for histological analyses. At gestational day 12.5, or 15.5, mothers were sacrificed immediately after moving them from the hypoxic chamber to maintain hypoxic conditions in order to dissect embryonic hearts for RNA isolation or histological analyses. All animal experiments were performed with approval from the University of Florida Institutional Animal Care and Use Committee.

### RNA-seq and real-time reverse transcriptase (RT)-PCR

Total RNAs were isolated from E12.5 wild-type and mutant hearts with or without hypoxia. To maintain hypoxic conditions, hearts were dissected immediately, snap frozen, and stored at −80°C until RNA isolation. To obtain enough RNA, two to three hearts were combined to prepare a single RNA sample (*N* = 3 or 4 samples from a total of 8–12 hearts were analyzed for each group). RNA library preparations and sequences were performed at MIT Genome Technology Core (Cambridge, MA). All RNA samples had a RNA integrity number above 8, which was considered high quality. Poly-adenylated RNA via oligo dT purification was utilized for library preparation via standard TruSeq protocol (Illumina, San Diego, CA). Reads were single-end and 40 bases pairs long each. The sequence depth was approximately 35 million reads per sample.

Analysis of RNA sequences was performed as follows: first, the quality of the sequence and data was checked using FastQ to detect over-represented K-mers, GC content, and the presence of adaptors. We indexed the MM9 genome from Ensembl using Bowtie2. The raw reads were mapped to the MM9 genome using Tophat. A majority (75–85%; average 82%) of the reads were successfully mapped according to Samstat. After mapping, the gene expression was quantified with Cufflinks software, which normalizes transcript length, number of reads, and sequencing biases by calculating FPKM values (fragments per kilobase of exon model per million mapped reads). A *P*-value < 0.05 and a fold change of more than 2 relative to control wild-normoxia hearts were considered significant. The resulting matrices were reordered based on their expression values using the Euclidean distance and Single Linkage clustering methods, and heatmaps of the differential expression values (in log2 scale) were generated using the Permut Matrix program.

RT-PCR was performed using inventoried TaqMan gene expression assays (Applied Biosystems, Foster City, CA): Nkx2-5 Mm00657783, Hey2 Mm00469280, Scn5a Mm00451971, KcneI Mm01215533, ANF Mm01255748, and hopx/HOD Mm00558629. Data were normalized to ß-actin expression (no. 4352933E). Duplicate experiments were averaged.

### Histological analysis and western blotting

Serial paraffin-embedded tissue sectioning of 5 μm thickness was performed as described previously (13). Two observers examined the digitalized images of the sections and performed quantitative histological measurements of trabecular vs. compact area size using the same analytical methods with Image J as reported previously (13). Immunostaining and Western blotting were performed with the following primary antibodies or a cell death detection system: Nkx2-5 pAb (14), GAPDH (MAB374, Millipore, Bedford, MA), phospho-histone H3 (serine 10; Millipore 06-570), and TUNEL (*In Situ* Cell Death Detection kit, Roche, Basel, Switzerland). Fluorescent microscopic images were obtained using a Axiovert200M (ZEISS, Oberkochen, Germany) attached to CCD camera. Digitalized images were utilized for measurement using Image J software as described (15–17).

### Alkaline phosphatase treatment of Nkx2-5

To maintain hypoxic conditions, hearts were dissected immediately, snap frozen, and stored at −80°C until protein purification. After rinsing with Tris-buffered saline, E12.5 hearts were briefly sonicated in the phosphatase buffer (50 mM Tris pH 9.3, 1 mM MgCl_2_, 0.1 mM ZnCl_2_, 10% glycerol, 1 mM dithiothreitol, 1 mM phenylmethylsulfonyl fluoride), and centrifuged. Ten unit of calf intestinal alkaline phosphatase (CIAP, New England BioLabs, Ipswich, MA) was added to the 20 μl of supernatant and incubated for 30 min at 30°C. As controls, the sample reaction was performed in the presence of 20 mM Na_2_HPO_4_ to inhibit a phosphatase reaction.

### Statistical analysis

Data presented are expressed as mean values plus or minus the standard error of the mean. Results were analyzed by SPSS (version 22) using crosstabs with Fisher's exact test, non-parametric test, analysis of variance with Fisher's *post-hoc* test, or independent *T*-test. Levene's test was utilized for equality of variance, and *P*-values were calculated depending on the assurance of equality. *P*-values less than 0.05 were considered significant.

## Results

### Applying gestational hypoxia (14% O_2_) beginning from mid-gestation on wild-type and *Nkx2-5* mutant embryos

**Figure 1:**
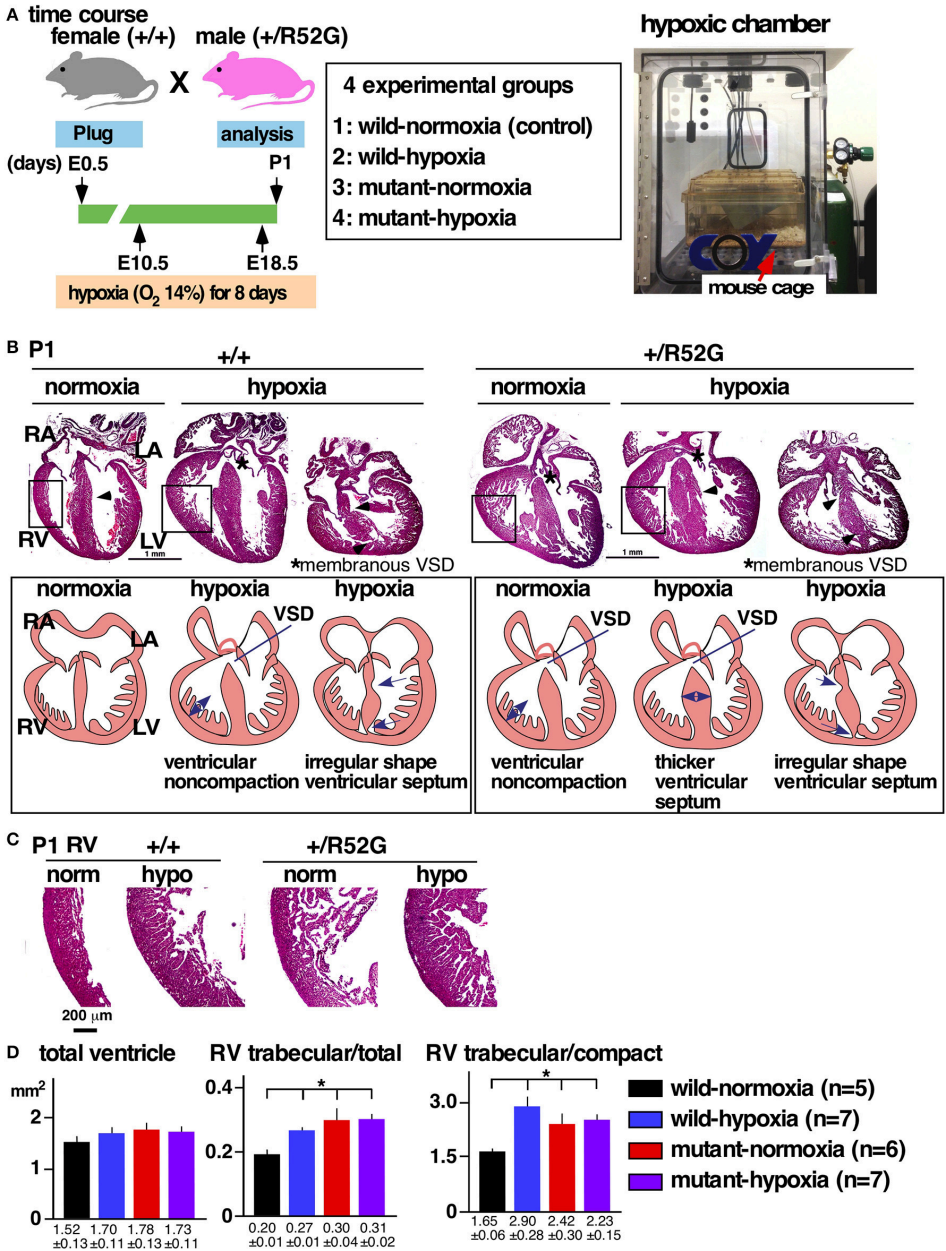
Experimental design of gestational hypoxia (14% O2 saturation) leading to cardiac anomalies that overlap with heterozygous *Nkx2-5* mutants. **(A)** Timelines of experiments and hypoxic chamber. **(B)** Representative images of P1 heart sections with simplified illustrations. Left, wild-type (+/+) normoxia and hypoxia; right, *Nkx2-5* mutant (+/R52G) normoxia and hypoxia. **(C)** Enlarged images of heart sections of the RV. **(D)** Quantification of area size of the total ventricle, RV trabecular layer relative to total ventricle, and RV trabecular relative to RV compact layer (mean ± S.E.). LA, left atrium; LV, left ventricle; RA, right atrium; RV, right ventricle; and VSD, ventricular septal defect. *P < 0.05.

To apply a physiological level of hypoxia, wild-type (+/+) female mice bred with heterozygous *Nkx2-5* mutant (+/R52G) male mice were housed in a 14% hypoxic chamber from 10.5 days of gestation, when pregnancy was evident. This mating allowed us to examine the effects of hypoxia on wild-type as well as heterozygous *Nkx2-5* mutant embryos without consideration of maternal cardiac defects due to a *Nkx2-5* mutation (Figure 1A). The mothers were moved out of the hypoxic chamber on gestational day 18.5 for delivery. Except for hypoxia, other conditions, such as a 12-h day-night cycle, temperature, and food, remained the same between the normoxia and hypoxia groups. Hereafter, the four groups of mice are referred to as wild-normoxia, wild-hypoxia, mutant-normoxia, and mutant-hypoxia.

### Cardiac anomalies in newborn wild-type and *Nkx2-5* mutant with or without gestational hypoxia

The newborn mouse hearts were fixed and examined for cardiac anomalies using 5-μm serial tissue sectioning of entire hearts as we described previously (8, 13, 18).

#### Overall cardiac anomalies displayed in both wild-hypoxia and Nkx2-5 mutant mice: ventricular septal defects (VSD), excessive ventricular trabeculation and irregular-shaped ventricular septum

Representative heart tissue sections obtained from P1 wild-normoxia, wild-hypoxia, mutant-normoxia, and mutant-hypoxia mice are shown in Figure 1B. In contrast to control wild-normoxia mice (*n* = 14) who did not show any cardiac anomalies, wild-hypoxia mice (*n* = 16) showed membranous or muscular VSDs, excessive ventricular trabeculation in the right ventricle (RV), and an irregular interventricular septum (Figure 1B, +/+ hypoxia; Table 1A). Of note, ventricular septal formation is completed with the interventricular communication being closed by embryonic day E13.5 to 14 in normal mouse embryos (19). The spectrum of cardiac anomalies displayed in wild-hypoxia mice overlaps with those in mutant-normoxia mice in this study (Figure 1B, +/R52G; Table 1B), similar to our previous study (8). In mutant mice, the penetrance of the VSD between the normoxia and hypoxia groups (73 vs. 79%), excessive ventricular trabeculation (both 100%), and irregular interventricular septum (87 vs. 86%) was not significantly different (Table 1C).

**Table 1A:** Cardiovascular malformations, wild-type normoxia vs. hypoxia.

	**W/W normoxia (*n* = 14)**	**W/W hypoxia (*n* = 16)**	***P* (WW normoxia vs. WW hypoxia)**	***X*^2^**
Any malformations	0 (0%)	12 (75%)	0.000^*^	20.1
Excessive trabeculation	0 (0%)	10 (63%)	0.000^*^	13.1
Ventricular septal defects	0 (0%)	4 (25%)	0.045^*^	5.3
Irregular-shaped ventricular septum	0 (0%)	9 (56%)	0.000^*^	15.2

* *P < 0.05*.

**Table 1B:** Cardiovascular malformations, wild-type vs. *Nkx2-5* mutant (W/R52G) at normoxia.

	**W/W normoxia (*n* = 14)**	**W/R52G normoxia (*n* = 15)**	***P* (WW normoxia vs. W/R52G normoxia)**	***X*^2^**
Any malformations	0 (0%)	15 (100%)	0.000^*^	29.0
Excessive trabeculation	0 (0%)	15 (100%)	0.000^*^	29.0
Ventricular septal defects	0 (0%)	11 (73%)	0.000^*^	16.5
Irregular-shaped ventricular septum	0 (0%)	13 (87%)	0.000^*^	22.0

* *P < 0.05*.

**Table 1C:** Cardiovascular malformations, *Nkx2-5* mutant normoxia vs. hypoxia.

	**W/R52G normoxia (*n* = 15)**	**W/R52G hypoxia (*n* = 14)**	***P*(W/R52G normoxia vs. W/R52G hypoxia)**	***X*^2^**
Any malformations	15 (100%)	14 (100%)	1.0	NA^#^
Excessive trabeculation	15 (100%)	14 (100%)	1.0	NA^#^
Ventricular septal defects	11 (73%)	11 (79%)	1.0	0.109
Irregular-shaped ventricular septum	13 (87%)	12 (86%)	1.0	0.006

# *Since all the animals had any malformations including ventricular noncompaction, X^2^ cannot be calculated, however there were no statistical differences between two groups (Fisher's test P = 1.0)*.

#### Excessive ventricular trabeculation

The ventricular wall is composed of an outer compact layer and an inner trabecular layer. The representative enlarged images of RV showed a thickened trabecular layer with deep intertrabecular recesses (20) in wild-hypoxia and mutants compared to control wild-normoxia (Figure 1C). We quantified the total area of the trabecular and compact layers and compared the relative ratio to that of randomly selected newborn hearts by two independent observers using the constant criteria as shown in our previous study (8) throughout the analyses (*n* = 5–7, Figure 1D). A significant increase in the ratio of the RV trabecular layer relative to the total ventricle or the RV compact layer was found in wild-hypoxia and mutants compared to controls.

#### No changes in expression of proliferation and apoptosis markers

**Figure 2:**
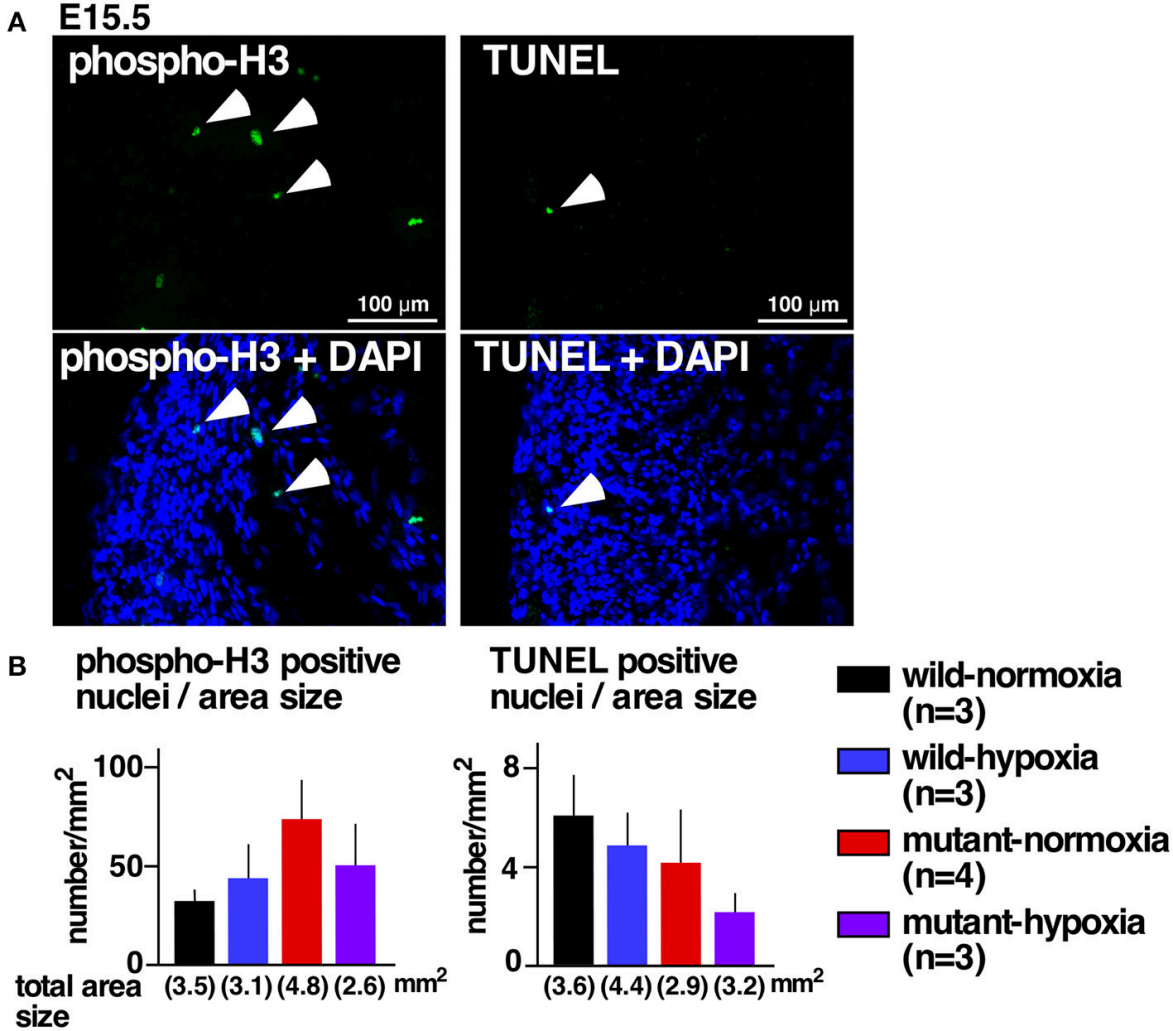
No changes in expression of proliferation and apoptosis markers. **(A)** Representative images of phospho-histone 3 staining (left) and TUNEL staining of RV wall tissue sections (right). Arrowheads indicate positively stained cells. **(B)** Summarized data (mean ± S.E.) of phospho-histone 3 positive nuclei and TUNEL positive nuclei in entire hearts relative to the area size obtained from multiple hearts. Total area size examined in each group is shown.

Excessive ventricular trabeculation may be related to abnormal cellular death or proliferations (21, 22). A number of cells positive for the cellular proliferation marker serine 10-phosphorylated histone H3 or the cell-death marker TUNEL, however, was not statistically different among the four groups (Figures 2A,B).

#### Atrial septum abnormalities

The most prevalent cardiac anomaly demonstrated in human patients with missense mutations in the *NKX2-5* homeodomain is atrial septal defects (6), characterized by persistent communications between the left and right atria, permitting postnatal shunting from left-to-right. During embryonic circulation, however, right-to-left shunting of blood through the foramen ovale is essential to circulate the oxygenated blood supplied from the maternal circulation. After birth, when the pulmonary circulation is established, the fossa ovalis is closed by attachment of the flap valve, in other words, the foreshortened primary septum, to its rims (23).

**Figure 3:**
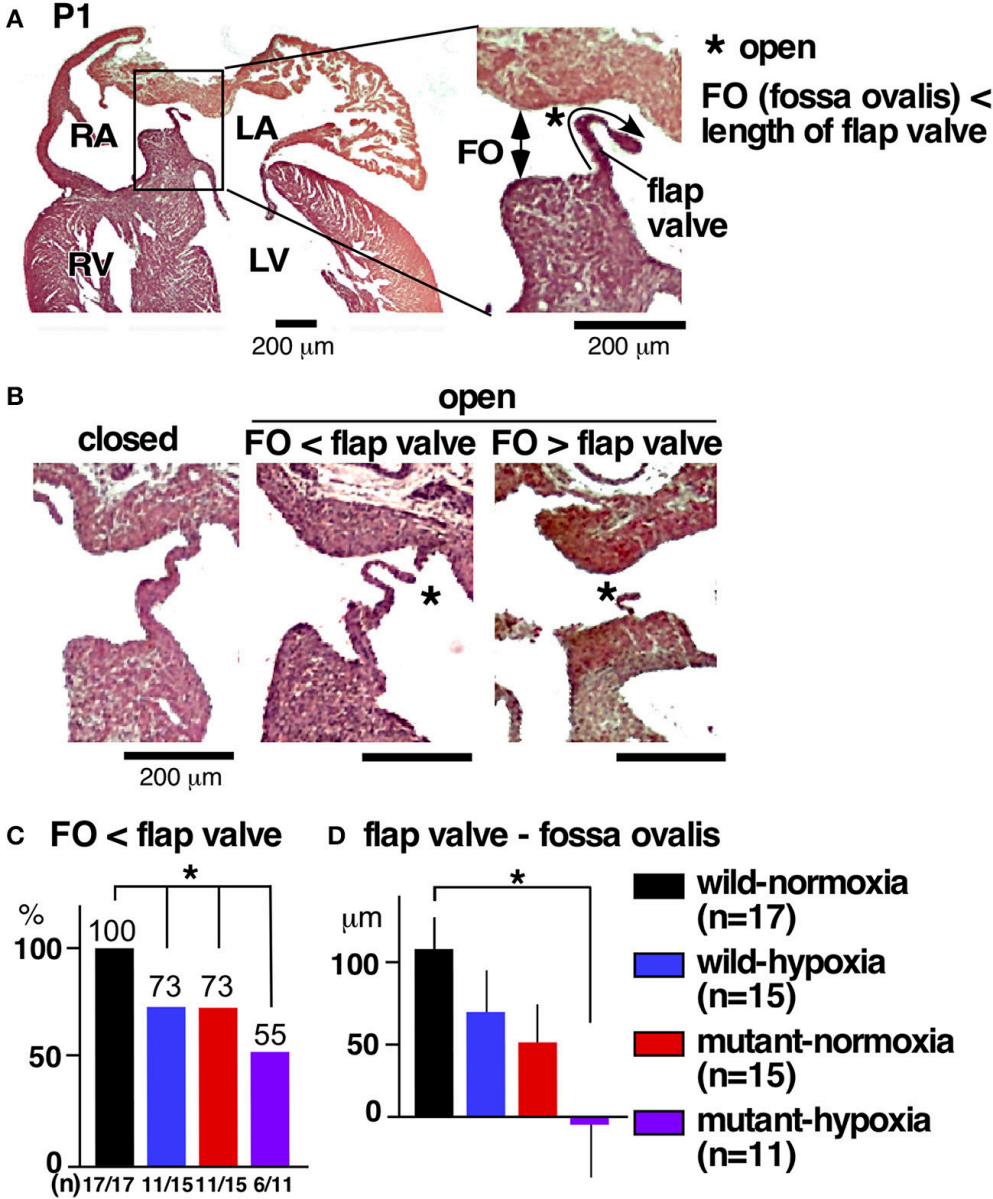
Comparison of interatrial communication on P1 hearts between three experimental groups and control (wild-normoxia). **(A)** Representative histological sections from P1 control hearts. Asterisk indicates open interactial communication. The size of fossa ovalis and the length of flap valve are indicated. **(B)** Representative histological sections from P1 hearts demonstrating closed fossa ovalis (left), open fossa ovalis where the size of fossa ovalis is smaller than the length of flap valve (middle), and open fossa ovalis where the size of the fossa ovalis is larger than the length of the flap valve (right). **(C)** Ratio of hearts showing the size of the fossa ovalis as smaller than the length of the flap valve. The number of mice examined is indicated. **(D)** The difference between the size of the fossa ovalis and the length of flap valve. *P < 0.05. FO, fossa ovalis.

During the transition between embryonic to postnatal circulation at P1, a majority of normal mice still demonstrated morphological interatrial communication, with a part of the fossa ovalis still not being sealed by the flap valve (Figure 3A). The size of the fossa ovalis, however, was smaller than the maximum length of the flap valve (Figure 3A), showing the potential that fossa ovalis will be physiologically sealed. Only a few mice showed attachment of the flap valve to the rim to close the fossa ovalis over the entire atrial septum (Figure 3B, closed). Quantitative analyses from a total of 58 hearts showed that size of the fossa ovalis was smaller than the maximum length of flap valve in all control hearts (wild-normoxia), which was reduced to 73% in wild-hypoxia and mutant-normoxia hearts, and further reduced to 55% in mutant-hypoxia hearts (Figure 3C). The difference between the length of the flap valve relative to the size of fossa ovalis, was highest in control wild-normoxia mice and was significantly reduced in mutant-hypoxia mice (Figure 3D, Supplemental Figure 1).

### Transcriptome overlaps between wild-hypoxia and *Nkx2-5* mutant-normoxia hearts

**Figure 4:**
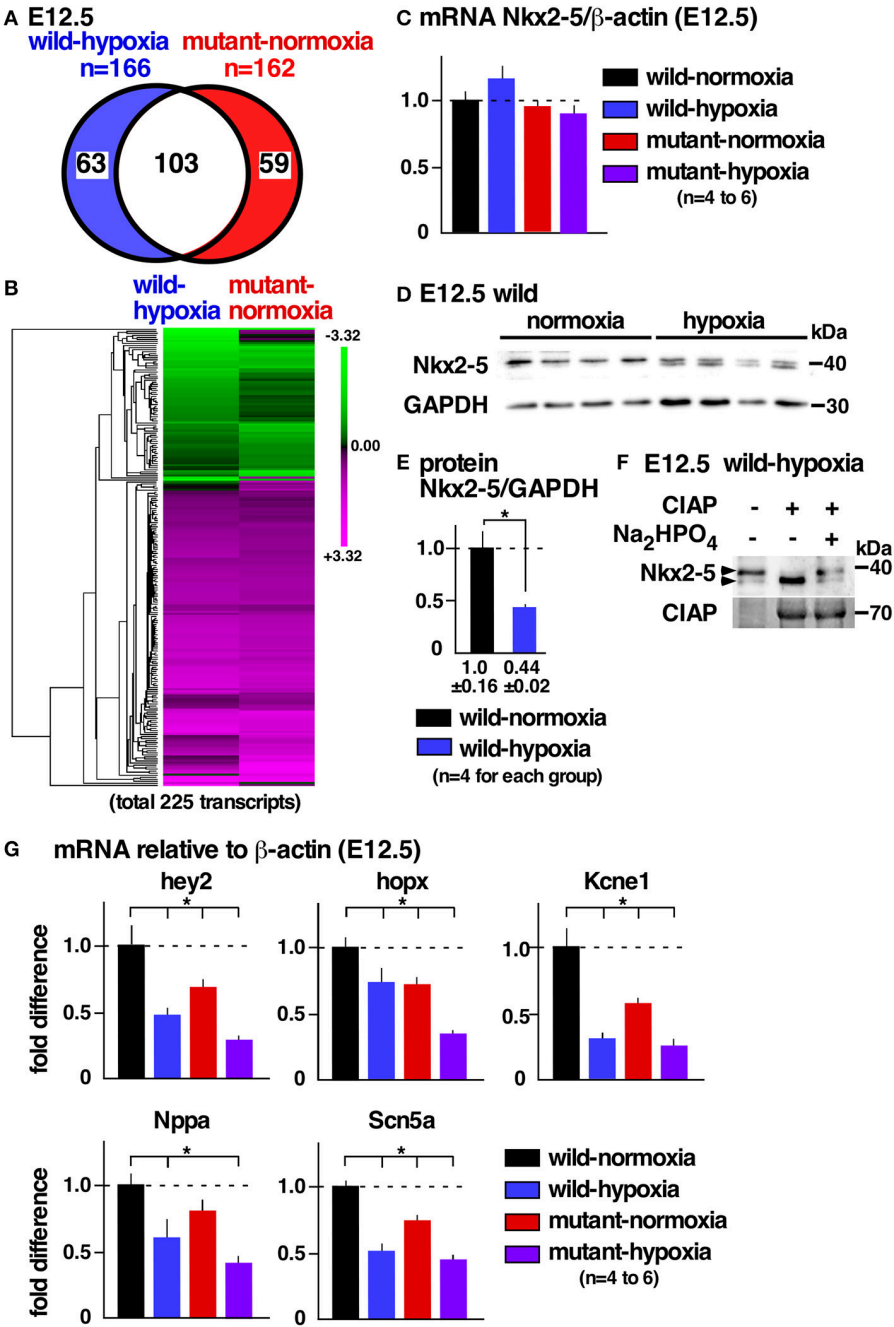
Gestational hypoxia led to a reduction of Nkx2-5 proteins and changes in mRNA expression overlapping with the *Nkx2-5* mutant. **(A)** RNA-seq data showed 166 genes in E12.5 wild-hypoxia hearts and 162 genes in normoxia *Nkx2-5* mutant hearts relative to the wild-normoxia hearts (*n* = 3 or 4 samples from each group). **(B)** The expression of 225 transcripts that were significantly changed in the wild-hypoxia hearts, or *Nkx2-5* mutant hearts, relative to control wild-normoxia hearts was visualized by heatmap exhibiting with log2 values. Upregulation (magenta), downregulation (green), and mean gene expression (black). **(C)** Real-time RT-PCR of Nkx2-5 mRNA relative to ß-actin in E12.5 hearts from four groups. **(D)** Western blotting demonstrating Nkx2-5 and GAPDH proteins in wild-normoxia and wild-hypoxia hearts. **(E)** Quantitative data for Nkx2-5 protein expression relative to GAPDH. **(F)** Western blotting demonstrates that the addition of phosphatase (CIAP) resulted in shifting a higher molecular weight band to a lower molecular weight band, which was inhibited by the addition of Na_2_HPO_4_ in the reaction performed side-by-side. **(G)** Real-time RT-PCR demonstrates the expression of several known Nkx2-5 targets normalized to ß-actin in four groups. Mean ± S.E. *P < 0.05.

To examine potential mechanisms underlying cardiac anomalies caused by gestational hypoxia, we performed differential gene expression analysis of developing hearts 2 days after gestational hypoxia (E12.5) using RNA-seq. Relative to control wild-normoxia hearts, 168 genes were differentially expressed in wild-hypoxia hearts, and 162 genes were differentially expressed in mutant-normoxia hearts (Figure 4A, Supplemental Tables 1, 2). Approximately 60% genes overlapped between wild-hypoxia and mutant-normoxia hearts. The expression of 225 transcripts were significantly changed in the hypoxia hearts, or *Nkx2-5* mutant-normoxia hearts, relative to control wild-normoxia hearts. These differences were visualized by a heatmap exhibiting with log2 values (Figure 4B). With some exceptions, the clustering of the expression values showed overlapping trends between wild-hypoxia and mutant-normoxia. This suggests the presence of a common mechanism underlying the cardiac anomalies that result from gestational hypoxia and the heterozygous *Nkx2-5* mutation. Expression of Nkx2-5 mRNA was unchanged by gestational hypoxia using RNA-seq (wild-hypoxia vs. wild-normoxia, fold difference = 1.11, *P* = 0.23; mutant-hypoxia vs. mutant-normoxia, fold difference = 0.99, *P*-value 0.95) and Taqman qRT-PCR (Figure 4C).

### Reduction of Nkx2-5 proteins in wild-type embryos with gestational hypoxia

Expression of Nkx2-5 protein, however, was reduced nearly one-half by gestational hypoxia relative to controls (Figures 4D,**E**). The majority of Nkx2-5 proteins from control hearts appeared to migrate into a single higher molecular weight. In contrast, Nkx2-5 proteins were separated into two distinct bands by hypoxia (Figure 4D), due to a reduction in phosphorylation (Figure 4F). mRNA expression of several known downstream targets of Nkx2-5 was reduced in wild-hypoxia and mutant hearts (Figure 4G). To note, Nkx2-5 acts not only as an activator but also as a repressor depending on the context of the target genes in mouse mid-embryonic hearts (13).

The expression of mRNA or protein of several important cardiac transcription factors, including Tbx5, Gata4, Mef2c, Hand1 and 2, was examined in E12.5 hearts by RNA-seq or Western blotting. The expression of these transcription factors was unchanged by gestational hypoxia or the heterozygous *Nkx2-5* mutant (Supplemental Table 3, Supplemental Figure 2).

Overall, there were phenotypic similarities between wild-hypoxia and *Nkx2-5* mutant hearts, and Nkx2-5 proteins were reduced nearly by one-half after gestational hypoxia.

## Discussion

Although gestational hypoxia and a mutation in *Nkx2-5* in mice have been shown to cause congenital heart disease (8, 24, 25), we report for the first time to our knowledge that non-genetic and genetic cardiac anomalies share a common mechanism relating to abnormal function of Nkx2-5, at least at a specific developing stage, E12.5. Moderate gestational hypoxia, namely 14% oxygen saturation, induces cardiac anomalies in wild-type mice, such as VSDs, excessive ventricular trabeculation, and irregular interventricular septum morphology. The spectrum of these cardiac anomalies overlaps with cardiac anomalies genetically induced by a heterozygous *Nkx2-*5 mutation in mice and humans. Gestational hypoxia reduces the expression of the Nkx2-5 protein by nearly one-half, and a genome-wide screening of mRNA shows that approximately 60% of dysregulated genes are overlapped between wild-hypoxia and mutant-normoxia relative to controls. To note, a 50% reduction of Nkx2-5 in heterozygous knockout mice leads to cardiac anomalies, including VSDs and ASDs (8, 26–28). In our study, VSDs were observed in 33% of heterozygous *Nkx2-5* knockout mice (8), with this incidence in agreement with other studies (26–28), whereas VSDs were observed in 82% of heterozygous *Nkx2-5* knock-in mice having the same genetic background (8).

The expression of Nkx2-5 proteins was reduced in embryonic hearts after 2 days of hypoxia without changing mRNA expression. Under various environmental stressors including hypoxia, all organisms respond and defend themselves to survive (29). Because proteins catalyze most cellular processes, rapid changes in protein levels are critical. There are many post-transcriptional steps at which cellular protein levels can be regulated, including abnormal RNA processing such as exporting to the cytoplasm, mRNA processing, and localization, translation, and post-translational modifications of proteins, as well as protein degradation (29). Nkx2-5 proteins are highly phosphorylated in hearts *in vivo* (30), but were reduced after exposure to hypoxia, suggesting that protein modifications, including phosphorylation, may be involved. There are open questions as to whether a reduction of the Nkx2-5 protein is a defensive mechanism in cardiac development, and how it is reduced under hypoxia.

During normal cardiac development, the ventricular trabecular layer is formed on approximately E10.5, coinciding with an increase of in the ventricular myocardial mass (31, 32), likely to facilitate the exchange of oxygen and nutrients from the blood locating in the ventricular cavities. As normal cardiac development progresses, development of discrete coronary arteries allows the outer compact layers to thicken, with the trabecular layer becoming less obvious, but this is altered during gestational hypoxia. Increased trabeculation, or ventricular noncompaction, is a cardiomyopathy with persistence of the trabecular layer, which can lead to both diastolic and systolic dysfunction (20, 33–35). Mechanisms leading to excessive ventricular trabeculation by gestational hypoxia and the *Nkx2-5* mutation need further investigation. For instance, excessive ventricular trabeculation was more evident in the right ventricle compared to the left by gestational hypoxia in P1 hearts. The reduction in Nkx2-5 proteins, however, was not apparently different between right and left ventricles in E12.5 hearts (Supplemental Figure 3). This might suggest that the hypoxia on heart anomalies may involve other factors, such as Isl1, acting in the secondary heart field (36).

RNAseq was examined after 2 days of hypoxia with the intention of finding early responses that lead to cardiac anomalies without being affected by complex compensatory mechanisms. Nevertheless, a limited number of transcripts, namely about 150 genes, were differentially expressed relative to control wild-normoxia; our study could have missed initial responses that occured earlier than 2 days of hypoxia. Alternatively, this stage may be too early to explain the cardiac phenotypes examined in P1 hearts. This is the same for heterozygous *Nkx2-5* mutant mice in which cardiac development will be most likely affected prior to E12.5.

Our study agrees with the previous studies showing that gestational hypoxia causes cardiac anomalies when exposed to lower oxygen levels. For instance, 10.5% oxygen saturation between E10.5 and E13.5 led to VSDs with an incidence of 33% (*n* = 5 out of 15 mice) and ventricular non-compaction (24). The susceptibility of mouse hearts to hypoxia appeared stage-dependent, in which E10.5 embryos have the highest susceptibility between E10.5 and E18.5 (24). Outflow tract anomalies, such as double outlet right ventricle, were displayed under ~10.5% hypoxia (24); but not in our hypoxic conditions using the 129/Sv mouse strain. Extreme hypoxia, namely 5.5% oxygen saturation for 8 h, led mice with the C57BL/6 background to cardiac anomalies (25). However, this low level of hypoxia will not be applicable, even on the highest mountain, Mt. Everest (22,800 feet or 6,960 meters, 6.9% oxygen saturation), and adult mice died under 5.5% oxygen saturation within 1 to 2 h under our experimental conditions using the 129/Sv mouse strain.

Under extreme hypoxia, such as 1–5% O2 in cell culture, transcription factor hypoxia-induced factor 1 alpha (Hif1α) protein is stabilized and its short half-life is extended (36–38). Several studies showed Hif1α plays a critical role in cardiac development with induction of Nkx2-5 transcription using Hif1α knockdown, knockout or cobalt chloride, which elicits hypoxia-like responses (36, 39, 40). Under 14% hypoxia starting from E10.5, however, there were no changes in expression of Nkx2-5 mRNA 2 days and 8 days of exposure of hypoxia, or Hif1α protein within 8 days of exposure of hypoxia relative to the age-matched normoxic condition (Supplemental Figure 4).

We introduced hypoxia beginning at gestation day 10.5, when pregnancy was confirmed by weight gain (12) and abdominal expansion. In humans, increased levels of human chorionic gonadotropin is a sensitive marker for pregnancy that is detectable shortly after pregnancy occurs, however, these tests for mice are not currently available to the research (12).

We initiated this study to test whether the severity of genetic cardiac anomalies induced by the heterozygous *Nkx2-5* mutation will be worsened by a combination of genetic and environmental effects (41), i.e., gestational hypoxia. Expression of several *Nkx2-5* downstream targets were affected by gestational hypoxia, however, there was no significant difference in the incidence of cardiac anomalies, including VSDs, which was demonstrated in 73% of mutant-normoxia mice (*n* = 15) and 79% of mutant-hypoxia mice (*n* = 14).

In summary, we report that moderate chronic gestational hypoxia leads to cardiac anomalies that overlap with heterozygous *Nkx2-5* mutant mice accompanied by a reduction in Nkx2-5 proteins. Non-genetic and genetic cardiac anomalies share a common mechanism regarding the abnormal function of Nkx2-5. Just as important, this finding is likely to provide insights into the common molecular mechanisms underlying non-genetic and genetic cardiac anomalies. Such insights would potentially allow for the future development of specific therapeutic strategies for patients suffering from a wide-ranges of congenital cardiac anomalies.

## Author contributions

Experiments were designed and performed by OM, RC, CD, NP, MH, TG, JD, AR, and HK. The manuscript was prepared by OM and HK.

### Conflict of interest statement

The authors declare that the research was conducted in the absence of any commercial or financial relationships that could be construed as a potential conflict of interest.
