# Supplementary material for: Mechanism Sharing Between Genetic and Gestational Hypoxia-Induced Cardiac Anomalies
Source: Front Cardiovasc Med. 2018 Aug 13;5:100. doi: 10.3389/fcvm.2018.00100 (PMC6099185; doi:10.3389/fcvm.2018.00100)
Supplement: Supplementary file 1 [file Data_Sheet_1.PDF]

SUPPLEMENTAL MATERIAL

Supplement Table 1. mRNA expression between wild-hypoxia relative to wild-normoxia.

gene_id	gene	locus	value wild-hypoxia	value wild-normoxia	log2(fold_change)	p_value	q_value	significant
ENSMUSG00000001348	Acp5	9:22117327-22137756	38.4706	18.0341	1.09303	5.00E-05	0.00395438	yes
ENSMUSG00000001542	Ell2	13:75707483-75772356	8.54468	5.13736	0.733999	5.00E-05	0.00395438	yes
ENSMUSG00000001763	Tspan33	6:29694221-29718559	15.4282	7.73522	0.996054	5.00E-05	0.00395438	yes
ENSMUSG00000003849	Nqo1	8:107388224-107403206	3.97744	1.91776	1.05241	0.0005	0.0263692	yes
ENSMUSG00000004552	Ctse	1:131638305-131675505	39.7675	15.2975	1.37829	5.00E-05	0.00395438	yes
ENSMUSG00000004961	Syt5	7:4539764-4547541	2.90548	1.15078	1.33616	0.0002	0.0125309	yes
ENSMUSG00000005161	Prdx2	8:84956609-84976542	809.58	518.447	0.642976	0.00035	0.0196257	yes
ENSMUSG00000005681	Apoa2	1:171225053-171226379	5.21974	12.9232	-1.30791	0.00065	0.0322482	yes
ENSMUSG00000007659	Bcl2l1	2:152780667-152831728	94.6541	50.7183	0.90016	5.00E-05	0.00395438	yes
ENSMUSG00000008843	Cldn13	5:134914248-134915526	9.00175	3.31269	1.4422	5.00E-05	0.00395438	yes
ENSMUSG00000015342	Xk	X:9272755-9313250	5.41617	3.62387	0.579742	0.00035	0.0196257	yes
ENSMUSG00000016255	Tubb1	2:174450694-174457882	13.1918	6.2689	1.07336	5.00E-05	0.00395438	yes
ENSMUSG00000016526	Dyrk3	1:131127454-131138340	8.9429	3.37227	1.40702	5.00E-05	0.00395438	yes
ENSMUSG00000017677	Wsb1	11:79239371-79254671	50.3133	84.9159	-0.755095	5.00E-05	0.00395438	yes
ENSMUSG00000020029	Nudt4	10:95547006-95564167	142.147	84.1784	0.755862	5.00E-05	0.00395438	yes
ENSMUSG00000020034	Tcp11l2	10:84576625-84614359	12.806	7.41769	0.787775	5.00E-05	0.00395438	yes
ENSMUSG00000020142	Slc1a4	11:20302179-20332713	10.0885	6.43371	0.648992	5.00E-05	0.00395438	yes
ENSMUSG00000020609	Apob	12:7977647-8016835	0.840634	2.07735	-1.30519	0.00095	0.043331	yes
ENSMUSG00000020638	Cmpk2	12:26469203-26479835	3.47881	1.90909	0.865707	0.0001	0.0071814	yes
ENSMUSG00000020656	Grhl1	12:24572286-24617391	1.91933	3.88046	-1.01563	5.00E-05	0.00395438	yes
ENSMUSG00000020802	Ube2o	11:116537739-116581447	23.7623	13.5244	0.813113	0.0001	0.0071814	yes
ENSMUSG00000020908	Myh3	11:67078299-67102291	0.748388	0.246642	1.60136	5.00E-05	0.00395438	yes
ENSMUSG00000021061	Sptb	12:76580487-76710547	22.1366	14.9683	0.564527	0.0003	0.0174098	yes
ENSMUSG00000021102	Glrx5	12:105032688-105040910	118.494	81.027	0.54834	0.0001	0.0071814	yes
ENSMUSG00000021118	Plek2	12:78888690-78906964	7.5496	2.99058	1.33597	5.00E-05	0.00395438	yes
ENSMUSG00000021194	Chga	12:102554968-102565027	1.69037	0.215513	2.97149	5.00E-05	0.00395438	yes
ENSMUSG00000021268	Meg3	12:109541000-109571726	73.1426	142.349	-0.960647	5.00E-05	0.00395438	yes
ENSMUSG00000021508	Cxcl14	13:56288646-56296551	12.8363	3.39378	1.91926	5.00E-05	0.00395438	yes

ENSMUSG00000021704	Mtx3	13:92844786-92858230	3.02312	4.49963	-0.573766	0.0005	0.0263692	yes
ENSMUSG00000022099	Dmtn	14:70602183-70636048	9.84647	5.03206	0.968456	5.00E-05	0.00395438	yes
ENSMUSG00000022100	Xpo7	14:70654245-70778495	14.6316	9.90437	0.562952	0.00015	0.00997772	yes
ENSMUSG00000022228	Zscan26	13:21421274-21453730	11.3236	17.7618	-0.649449	0.0002	0.0125309	yes
ENSMUSG00000022382	Wnt7b	15:85535438-85593708	2.86906	0.945963	1.60072	5.00E-05	0.00395438	yes
ENSMUSG00000022483	Col2a1	15:97975601-98004695	15.9413	23.3032	-0.547762	0.00025	0.0149904	yes
ENSMUSG00000022510	Trp63	16:25683762-25892102	1.69786	0.571783	1.57018	0.00085	0.0398647	yes
ENSMUSG00000023216	Epb42	2:121017890-121037072	54.3903	23.4658	1.21279	0.0001	0.0071814	yes
ENSMUSG00000023926	Rhag	17:40811125-40840754	8.04221	4.55872	0.818963	5.00E-05	0.00395438	yes
ENSMUSG00000024014	Pim1	17:29461895-29496111	22.4309	13.6681	0.714672	5.00E-05	0.00395438	yes
ENSMUSG00000024276	Zfp397	18:23954687-23964671	7.73409	11.34	-0.552114	0.00015	0.00997772	yes
ENSMUSG00000024383	Map3k2	18:32163088-32236751	1.78692	2.64022	-0.563184	0.00065	0.0322482	yes
ENSMUSG00000024391	Apom	17:35128996-35132050	1.04062	3.01584	-1.53512	0.0005	0.0263692	yes
ENSMUSG00000024588	Fech	18:64456549-64489066	82.196	46.9979	0.806473	5.00E-05	0.00395438	yes
ENSMUSG00000024661	Fth1	19:9982702-9985092	1687.69	892.687	0.918828	0.00015	0.00997772	yes
ENSMUSG00000024990	Rbp4	19:38116619-38125321	6.01773	14.6648	-1.28507	5.00E-05	0.00395438	yes
ENSMUSG00000025270	Alas2	X:150519518-150643878	259.504	77.2906	1.74739	5.00E-05	0.00395438	yes
ENSMUSG00000025503	Taldo1	7:141392198-141402968	115.046	82.9549	0.471814	0.00105	0.0466517	yes
ENSMUSG00000025889	Snca	6:60731574-60829855	43.7368	19.8482	1.13984	5.00E-05	0.00395438	yes
ENSMUSG00000026034	Clk1	1:58410188-58424066	65.3626	116.597	-0.834994	5.00E-05	0.00395438	yes
ENSMUSG00000026349	Ccnt2	1:127774163-127808061	10.7825	17.1573	-0.670137	0.00035	0.0196257	yes
ENSMUSG00000026442	Nfasc	1:132564689-132741797	0.117249	1.01497	-3.11378	5.00E-05	0.00395438	yes
ENSMUSG00000026459	Myog	1:134289988-134292548	3.34804	1.30075	1.36397	0.00035	0.0196257	yes
ENSMUSG00000026532	Spta1	1:174172775-174248450	9.18516	5.76392	0.672255	5.00E-05	0.00395438	yes
ENSMUSG00000026686	Lmx1a	1:167689236-167848741	8.32641	3.62363	1.20026	5.00E-05	0.00395438	yes
ENSMUSG00000026688	Mgst3	1:167371965-167393841	73.0739	52.577	0.474925	0.00075	0.0361136	yes
ENSMUSG00000027077	Smtnl1	2:84811175-84822652	0.970164	0.291743	1.73353	0.00055	0.0283209	yes
ENSMUSG00000027078	Ube2l6	2:84798827-84810335	168.123	66.5527	1.33695	5.00E-05	0.00395438	yes
ENSMUSG00000027495	Fam210b	2:172345564-172355749	12.8166	7.00717	0.871112	5.00E-05	0.00395438	yes
ENSMUSG00000027510	Rbm38	2:173020497-173034734	102.597	70.6327	0.538581	0.00025	0.0149904	yes
ENSMUSG00000027562	Car2	3:14886272-14900770	176.707	74.0702	1.25439	5.00E-05	0.00395438	yes
ENSMUSG00000027620	Rbm39	2:156147238-156180238	130.029	201.081	-0.628943	0.0008	0.0378933	yes
ENSMUSG00000027677	Ttc14	3:33799831-33844310	10.5132	17.659	-0.748204	0.00035	0.0196257	yes

ENSMUSG00000027869	Hsd3b6	3:98805503-98814443	173.087	49.5985	1.80313	5.00E-05	0.00395438	yes
ENSMUSG00000027871	Hsd3b1	3:98852193-98859794	5.05442	2.07114	1.28712	5.00E-05	0.00395438	yes
ENSMUSG00000028081	Rps3a1	3:86137939-86142702	10.6923	3.73903	1.51584	5.00E-05	0.00395438	yes
ENSMUSG00000028393	Alad	4:62509168-62519918	96.0559	59.6683	0.686911	5.00E-05	0.00395438	yes
ENSMUSG00000028644	Ermap	4:119175456-119190011	23.6672	11.3026	1.06624	0.00025	0.0149904	yes
ENSMUSG00000028716	Pdzk1ip1	4:115088707-115093899	84.6945	30.0747	1.49372	5.00E-05	0.00395438	yes
ENSMUSG00000028717	Tal1	4:115056425-115071755	26.5037	14.4638	0.873752	5.00E-05	0.00395438	yes
ENSMUSG00000028730	Cfap57	4:118554550-118620777	1.68916	0.570909	1.56498	5.00E-05	0.00395438	yes
ENSMUSG00000028825	Rhd	4:134864535-134896172	18.058	8.46361	1.0933	5.00E-05	0.00395438	yes
ENSMUSG00000029068	Ccnl2	4:155812488-155824543	27.6705	46.6616	-0.753887	5.00E-05	0.00395438	yes
ENSMUSG00000029826	Zc3hav1	6:38305285-38354603	8.47249	5.24541	0.691732	5.00E-05	0.00395438	yes
ENSMUSG00000029922	Mkrn1	6:39397803-39421294	50.3515	25.5015	0.981451	5.00E-05	0.00395438	yes
ENSMUSG00000030000	Add2	6:86028680-86124409	27.8941	11.133	1.32512	5.00E-05	0.00395438	yes
ENSMUSG00000030406	Gipr	7:19156060-19166127	2.99637	5.42204	-0.855623	0.00025	0.0149904	yes
ENSMUSG00000030878	Cdr2	7:120957035-120982312	35.7881	21.4777	0.736638	5.00E-05	0.00395438	yes
ENSMUSG00000031762	Mt2	8:94170747-94173568	44.3586	23.4717	0.918294	0.0009	0.0416496	yes
ENSMUSG00000032083	Apoa1	9:46228579-46230466	7.26331	16.7289	-1.20364	5.00E-05	0.00395438	yes
ENSMUSG00000032449	Slc25a36	9:97074960-97111157	17.4454	30.3258	-0.797695	5.00E-05	0.00395438	yes
ENSMUSG00000032656	3-Mar	18:56761715-56925548	16.8955	8.83712	0.934992	5.00E-05	0.00395438	yes
ENSMUSG00000032715	Trib3	2:152337421-152344032	9.12755	3.47428	1.39351	5.00E-05	0.00395438	yes
ENSMUSG00000033006	Sox10	15:79154912-79164490	1.43843	0.625371	1.20171	0.0007	0.0342417	yes
ENSMUSG00000033214	Slitrk5	14:111675114-111683134	7.39255	11.0012	-0.573516	0.00015	0.00997772	yes
ENSMUSG00000033831	Fgb	3:83040140-83049863	0.294022	1.18989	-2.01683	5.00E-05	0.00395438	yes
ENSMUSG00000034160	Ogt	X:101640059-101684351	31.2369	48.471	-0.633872	0.0001	0.0071814	yes
ENSMUSG00000034248	Slc25a37	14:69241847-69305355	101.993	50.1535	1.02404	5.00E-05	0.00395438	yes
ENSMUSG00000034449	Dhrs11	11:84820855-84828994	20.7401	13.8264	0.584999	0.0008	0.0378933	yes
ENSMUSG00000035557	Krt17	11:100256216-100261029	2.93591	0.672897	2.12535	5.00E-05	0.00395438	yes
ENSMUSG00000036306	Lzts1	8:69132668-69184225	0.844946	7.89841	-3.22463	5.00E-05	0.00395438	yes
ENSMUSG00000036390	Gadd45a	6:67035095-67080654	18.1162	9.26594	0.967271	0.00025	0.0149904	yes
ENSMUSG00000037124	Trim58	11:58640464-58652404	2.04015	0.755652	1.43288	5.00E-05	0.00395438	yes
ENSMUSG00000037254	Itih2	2:10094592-10131396	0.994339	3.33738	-1.74691	5.00E-05	0.00395438	yes
ENSMUSG00000037415	Ranbp10	8:105768307-105827350	13.1461	9.34503	0.492364	0.00055	0.0283209	yes
ENSMUSG00000038059	Smim3	18:60474192-60501983	7.59547	4.69131	0.695149	0.0007	0.0342417	yes

ENSMUSG00000038871	Bpgm	6:34476206-34505613	162.162	65.5602	1.30654	5.00E-05	0.00395438	yes
ENSMUSG00000038893	Fam117a	11:95336010-95384507	28.0361	13.7706	1.02569	0.0001	0.0071814	yes
ENSMUSG00000039956	Mrap	16:90738323-90749785	4.73625	2.08791	1.18169	0.00015	0.00997772	yes
ENSMUSG00000040466	Blvrb	7:27447977-27466144	131.024	67.3931	0.959156	5.00E-05	0.00395438	yes
ENSMUSG00000040713	Creg1	1:165763745-165775308	130.609	68.48	0.931503	0.0001	0.0071814	yes
ENSMUSG00000041842	Fhdc1	3:84442197-84480429	2.28239	0.664453	1.78031	0.00095	0.043331	yes
ENSMUSG00000042066	Tmcc2	1:132356314-132391281	102.663	40.8468	1.32962	5.00E-05	0.00395438	yes
ENSMUSG00000042351	Grap2	15:80623504-80650559	13.9886	5.75261	1.28197	5.00E-05	0.00395438	yes
ENSMUSG00000044337	Ackr3	1:90203979-90216751	49.6784	32.5207	0.611259	5.00E-05	0.00395438	yes
ENSMUSG00000044468	Fam46c	3:100451627-100489324	29.4086	9.43641	1.63993	5.00E-05	0.00395438	yes
ENSMUSG00000045409	Trim39	17:36258872-36272247	5.19407	7.86547	-0.598669	0.00055	0.0283209	yes
ENSMUSG00000045545	Krt14	11:100203161-100207548	8.85173	1.28364	2.78572	5.00E-05	0.00395438	yes
ENSMUSG00000045573	Penk	4:4133530-4188703	15.1923	8.39983	0.854911	0.0001	0.0071814	yes
ENSMUSG00000045730	Adrb2	18:62177816-62179959	3.95644	1.74278	1.18281	5.00E-05	0.00395438	yes
ENSMUSG00000046352	Gjb2	14:57098599-57104702	4.85322	1.98971	1.28638	5.00E-05	0.00395438	yes
ENSMUSG00000047281	Sfn	4:133600555-133602168	1.94933	0.518232	1.91131	5.00E-05	0.00395438	yes
ENSMUSG00000048540	Nhlh2	3:102010075-102015492	0.170436	1.17596	-2.78653	5.00E-05	0.00395438	yes
ENSMUSG00000049409	Prokr1	6:87578590-87590743	3.02244	1.31427	1.20146	0.0011	0.0483395	yes
ENSMUSG00000050334	C130071C03Rik	13:83721380-83884194	0.173799	0.644235	-1.89017	0.00055	0.0283209	yes
ENSMUSG00000051251	Nhlh1	1:172052291-172057573	0.0986256	0.475507	-2.26943	0.0007	0.0342417	yes
ENSMUSG00000051451	Crebzf	7:90442728-90447994	22.0571	36.6036	-0.730744	0.00055	0.0283209	yes
ENSMUSG00000051839	Gypa	8:80493780-80510542	30.5112	14.9588	1.02834	5.00E-05	0.00395438	yes
ENSMUSG00000052217	Hbb-bh1	7:103841636-103843164	4555.82	1687.86	1.43251	5.00E-05	0.00395438	yes
ENSMUSG00000052305	Hbb-bs	7:103826533-103828096	4815.09	1116.87	2.1081	5.00E-05	0.00395438	yes
ENSMUSG00000053835	H2-T24	17:35994452-36042747	6.20525	3.05749	1.02114	0.00055	0.0283209	yes
ENSMUSG00000054146	Krt15	11:100131757-100135928	9.25719	1.49289	2.63247	5.00E-05	0.00395438	yes
ENSMUSG00000054191	Klf1	8:84901927-84905291	20.125	11.2714	0.836324	5.00E-05	0.00395438	yes
ENSMUSG00000054932	Afp	5:90490736-90515931	12.0074	45.781	-1.93083	5.00E-05	0.00395438	yes
ENSMUSG00000055401	Fbxo6	4:148145715-148152140	20.2871	9.96639	1.02542	5.00E-05	0.00395438	yes
ENSMUSG00000056656	Apol8	15:77747798-77755229	1.7654	0.645742	1.45097	0.00015	0.00997772	yes
ENSMUSG00000058620	Adra2b	2:127363207-127367221	4.55025	2.11821	1.1031	5.00E-05	0.00395438	yes
ENSMUSG00000058794	Nfe2	15:103248211-103258403	39.7201	15.9569	1.31569	5.00E-05	0.00395438	yes

ENSMUSG00000059481	Plg	17:12378608-12419384	0.394776	1.16188	-1.55735	0.0007	0.0342417	yes
ENSMUSG00000060257	Scrt2	2:152081528-152095802	0.203631	0.765603	-1.91064	0.0004	0.0219356	yes
ENSMUSG00000060807	Serpina6	12:103646629-103657212	4.48134	13.6952	-1.61166	5.00E-05	0.00395438	yes
ENSMUSG00000061527	Krt5	15:101707069-101712891	18.585	2.21267	3.07028	5.00E-05	0.00395438	yes
ENSMUSG00000063856	Gpx1	9:108338902-108340343	1523.46	707.008	1.10755	5.00E-05	0.00395438	yes
ENSMUSG00000069814	Ccdc92b	11:74619604-74641516	7.14617	2.27407	1.65189	5.00E-05	0.00395438	yes
ENSMUSG00000070803	Cited4	4:120666571-120667820	45.1231	27.3857	0.720445	5.00E-05	0.00395438	yes
ENSMUSG00000071068	Treml2	17:48300037-48312534	2.61497	1.26123	1.05196	5.00E-05	0.00395438	yes
ENSMUSG00000071711	Mpst	15:78406711-78414002	27.747	19.3182	0.522374	0.00095	0.043331	yes
ENSMUSG00000071715	Ncf4	15:78244800-78262580	30.6637	9.8877	1.63283	5.00E-05	0.00395438	yes
ENSMUSG00000071866	Ppia	11:6415442-6419817	1.09077	2.8906	-1.40602	5.00E-05	0.00395438	yes
ENSMUSG00000073063	Hbq1b	11:32286964-32287784	11.1511	3.54481	1.65341	5.00E-05	0.00395438	yes
ENSMUSG00000073400	Trim10	17:36869573-36877833	31.705	11.1214	1.51137	5.00E-05	0.00395438	yes
ENSMUSG00000073940	Hbb-bt	7:103812523-103813996	405.567	92.6762	2.12967	5.00E-05	0.00395438	yes
ENSMUSG00000074415	2610203C20Rik	9:41327259-41655483	4.42845	8.88406	-1.00442	5.00E-05	0.00395438	yes
ENSMUSG00000078853	Igtp	11:58199555-58222782	6.26394	3.34942	0.903159	0.0003	0.0174098	yes
ENSMUSG00000082101	Slfn14	11:83275109-83286726	6.73538	2.65499	1.34305	5.00E-05	0.00395438	yes
ENSMUSG00000082431	Tdpx-ps1	1:99772764-100485942	81.4281	52.2935	0.638896	5.00E-05	0.00395438	yes
ENSMUSG00000084289	Gm6977	X:90744545-90745062	538.237	284.331	0.920669	5.00E-05	0.00395438	yes
ENSMUSG00000084893	Hba-ps4	17:26286362-26287061	14.5319	4.25302	1.77267	0.0001	0.0071814	yes
ENSMUSG00000085006	BC021767	3:94661829-94670696	2.56557	0.784148	1.71008	5.00E-05	0.00395438	yes
ENSMUSG00000085396	Firre	X:50555743-50635321	13.7826	27.5323	-0.998278	0.0003	0.0174098	yes
ENSMUSG00000085700	Hbb-bh0	7:103850019-103850148	5984.43	1515.31	1.9816	5.00E-05	0.00395438	yes
ENSMUSG00000086804	Gm43154	6:31605189-31922270	1.61251	0.545471	1.56374	0.00035	0.0196257	yes
ENSMUSG00000089726	Mir17hg	14:115042878-115046727	1.62918	4.02602	-1.30521	5.00E-05	0.00395438	yes
ENSMUSG00000090877	Hspa1b	17:34956435-34959238	1.68833	0.51139	1.7231	5.00E-05	0.00395438	yes
ENSMUSG00000091971	Hspa1a	17:34969189-34972156	1.94894	0.56164	1.79497	5.00E-05	0.00395438	yes
ENSMUSG00000095041	AC149090.1	JH584304.1:52189-59690	2.03815	3.80533	-0.90076	0.00015	0.00997772	yes
ENSMUSG00000095123	Gm21781	10:4391586-4396424	0.502765	1.13733	-1.1777	0.00035	0.0196257	yes
ENSMUSG00000095139	Pou3f2	4:22482779-22488366	0.0671455	0.402906	-2.58508	5.00E-05	0.00395438	yes
ENSMUSG00000096768	Erdr1	Y:90784737-90816464	1.50272	4.54334	-1.59618	0.0002	0.0125309	yes
ENSMUSG00000097023	Mir9-3hg	7:79500025-79534403	0.182782	1.21183	-2.729	0.00015	0.00997772	yes

ENSMUSG00000097375	6720427I07Rik	13:48535522-48537822	2.49825	4.89576	-0.970615	5.00E-05	0.00395438	yes
ENSMUSG00000097767	Miat	5:112213227-112233720	0.140169	0.682396	-2.28344	0.00035	0.0196257	yes
ENSMUSG00000097977	Gm5652	12:5589616-5590624	0.559334	2.63252	-2.23466	5.00E-05	0.00395438	yes
ENSMUSG00000103953	Gm29718	1:136793033-136793510	43.0551	27.1993	0.662614	0.00105	0.0466517	yes
ENSMUSG00000107726	Gm44037	4:103217701-103219592	0.175312	0.721695	-2.04147	0.00065	0.0322482	yes
ENSMUSG00000108322	Gm45123	7:44484516-44485413	16.8645	29.7437	-0.818591	5.00E-05	0.00395438	yes
ENSMUSG00000108621	Gm37494	7:39542536-39580589	0.507958	1.53477	-1.59524	0.001	0.0449261	yes
ENSMUSG00000109706	Fth-ps3	8:86902452-86919422	43.7441	22.2895	0.972722	0.0004	0.0219356	yes

Supplement Table 2. mRNA expression between mutant-normoxia relative to wild-normoxia.

2gene_id	gene	locus	value mutant-normoxia	value wild-normoxia	log2(fold_change)	p_value	q_value	significant
ENSMUSG00000000094	Tbx4	11:85886421-85916097	1.58823	0.455615	1.80153	5.00E-05	0.00395438	yes
ENSMUSG00000001348	Acp5	9:22117327-22137756	31.1314	18.0341	0.787646	5.00E-05	0.00395438	yes
ENSMUSG00000001542	Ell2	13:75707483-75772356	8.60432	5.13736	0.744035	5.00E-05	0.00395438	yes
ENSMUSG00000001763	Tspan33	6:29694221-29718559	12.8244	7.73522	0.72938	5.00E-05	0.00395438	yes
ENSMUSG00000004552	Ctse	1:131638305-131675505	34.3435	15.2975	1.16674	5.00E-05	0.00395438	yes
ENSMUSG00000004885	Crabp2	3:87948665-87953376	21.2674	13.0415	0.70553	0.0008	0.0378933	yes
ENSMUSG00000004961	Syt5	7:4539764-4547541	2.60237	1.15078	1.17721	0.00055	0.0283209	yes
ENSMUSG00000005917	Otx1	11:21994763-22002897	1.04575	0.244387	2.09729	0.00025	0.0149904	yes
ENSMUSG00000007279	Scube2	7:109798675-109865679	4.95666	7.60219	-0.617047	0.00085	0.0398647	yes
ENSMUSG00000007659	Bcl2l1	2:152780667-152831728	84.2979	50.7183	0.732992	0.00015	0.00997772	yes
ENSMUSG00000007805	Twist2	1:91798527-91848028	6.68945	3.52604	0.92384	0.0006	0.0304166	yes
ENSMUSG00000008843	Cldn13	5:134914248-134915526	6.4308	3.31269	0.956996	0.0008	0.0378933	yes
ENSMUSG00000013584	Aldh1a2	9:71215788-71296243	19.6134	12.785	0.61739	5.00E-05	0.00395438	yes
ENSMUSG00000015342	Xk	X:9272755-9313250	6.11946	3.62387	0.755873	5.00E-05	0.00395438	yes
ENSMUSG00000016255	Tubb1	2:174450694-174457882	11.5953	6.2689	0.887258	5.00E-05	0.00395438	yes
ENSMUSG00000016526	Dyrk3	1:131127454-131138340	7.62271	3.37227	1.17658	5.00E-05	0.00395438	yes

ENSMUSG00000020029	Nudt4	10:95547006-95564167	133.278	84.1784	0.662914	5.00E-05	0.00395438	yes
ENSMUSG00000020034	Tcp11l2	10:84576625-84614359	11.5727	7.41769	0.641684	0.0001	0.0071814	yes
ENSMUSG00000020142	Slc1a4	11:20302179-20332713	10.696	6.43371	0.733346	5.00E-05	0.00395438	yes
ENSMUSG00000020638	Cmpk2	12:26469203-26479835	3.44989	1.90909	0.853663	0.00015	0.00997772	yes
ENSMUSG00000020656	Grhl1	12:24572286-24617391	2.38272	3.88046	-0.703619	0.00085	0.0398647	yes
ENSMUSG00000020802	Ube2o	11:116537739-116581447	21.2376	13.5244	0.651057	0.0001	0.0071814	yes
ENSMUSG00000020875	Hoxb9	11:96271456-96276595	0.954834	0.247345	1.94873	0.00065	0.0322482	yes
ENSMUSG00000020908	Myh3	11:67078299-67102291	0.713012	0.246642	1.53151	5.00E-05	0.00395438	yes
ENSMUSG00000020950	Foxg1	12:49382659-49407360	1.90848	0.361688	2.3996	5.00E-05	0.00395438	yes
ENSMUSG00000021061	Sptb	12:76580487-76710547	22.6996	14.9683	0.60076	0.00015	0.00997772	yes
ENSMUSG00000021118	Plek2	12:78888690-78906964	6.89315	2.99058	1.20474	5.00E-05	0.00395438	yes
ENSMUSG00000021255	Esrrb	12:86361116-86521628	1.66073	3.25131	-0.969203	5.00E-05	0.00395438	yes
ENSMUSG00000021381	Barx1	13:48662997-48666507	5.91099	0.909516	2.70023	5.00E-05	0.00395438	yes
ENSMUSG00000021508	Cxcl14	13:56288646-56296551	11.943	3.39378	1.8152	5.00E-05	0.00395438	yes
ENSMUSG00000021732	Fgf10	13:118669790-118792115	1.64257	0.558102	1.55735	0.00025	0.0149904	yes
ENSMUSG00000022099	Dmtn	14:70602183-70636048	9.00519	5.03206	0.839607	5.00E-05	0.00395438	yes
ENSMUSG00000022100	Xpo7	14:70654245-70778495	14.1625	9.90437	0.515944	0.00025	0.0149904	yes
ENSMUSG00000022309	Angpt1	15:42424726-42704616	20.055	29.5955	-0.561413	5.00E-05	0.00395438	yes
ENSMUSG00000022382	Wnt7b	15:85535438-85593708	3.80499	0.945963	2.00804	5.00E-05	0.00395438	yes
ENSMUSG00000022483	Col2a1	15:97975601-98004695	9.91691	23.3032	-1.23257	5.00E-05	0.00395438	yes
ENSMUSG00000022510	Trp63	16:25683762-25892102	1.98769	0.571783	1.79755	0.0001	0.0071814	yes
ENSMUSG00000023216	Epb42	2:121017890-121037072	49.8641	23.4658	1.08744	0.00045	0.0242455	yes
ENSMUSG00000023906	Cldn6	17:23679364-23682446	3.77394	1.4003	1.43033	0.00045	0.0242455	yes
ENSMUSG00000023926	Rhag	17:40811125-40840754	8.00356	4.55872	0.812013	5.00E-05	0.00395438	yes
ENSMUSG00000023995	Tspo2	17:48447955-48451522	4.83374	0.730603	2.72598	0.0006	0.0304166	yes
ENSMUSG00000024014	Pim1	17:29461895-29496111	23.2965	13.6681	0.769296	5.00E-05	0.00395438	yes
ENSMUSG00000024134	Six2	17:85684276-85688274	2.36758	0.798459	1.56812	5.00E-05	0.00395438	yes
ENSMUSG00000024479	Mal2	15:54571365-54602846	0.525719	0.0856595	2.61761	0.00015	0.00997772	yes
ENSMUSG00000024588	Fech	18:64456549-64489066	77.7599	46.9979	0.726431	5.00E-05	0.00395438	yes
ENSMUSG00000024661	Fth1	19:9982702-9985092	1581.61	892.687	0.825167	0.00075	0.0361136	yes
ENSMUSG00000024990	Rbp4	19:38116619-38125321	6.30135	14.6648	-1.21863	5.00E-05	0.00395438	yes
ENSMUSG00000025270	Alas2	X:150519518-150643878	192.299	77.2906	1.31499	5.00E-05	0.00395438	yes
ENSMUSG00000025407	Gli1	10:127323726-127341589	1.3952	0.606788	1.2012	0.0005	0.0263692	yes

ENSMUSG00000025889	Snca	6:60731574-60829855	40.6487	19.8482	1.0342	5.00E-05	0.00395438	yes
ENSMUSG00000026100	Mstn	1:53061639-53068079	4.75968	8.09066	-0.765394	5.00E-05	0.00395438	yes
ENSMUSG00000026459	Myog	1:134289988-134292548	3.14169	1.30075	1.2722	0.0003	0.0174098	yes
ENSMUSG00000026532	Spta1	1:174172775-174248450	10.4614	5.76392	0.859962	5.00E-05	0.00395438	yes
ENSMUSG00000026686	Lmx1a	1:167689236-167848741	7.74979	3.62363	1.09672	5.00E-05	0.00395438	yes
ENSMUSG00000027078	Ube2l6	2:84798827-84810335	142.95	66.5527	1.10294	5.00E-05	0.00395438	yes
ENSMUSG00000027168	Pax6	2:105536079-105697364	2.2606	0.604694	1.90243	0.00015	0.00997772	yes
ENSMUSG00000027495	Fam210b	2:172345564-172355749	11.5536	7.00717	0.72144	5.00E-05	0.00395438	yes
ENSMUSG00000027562	Car2	3:14886272-14900770	157.792	74.0702	1.09106	5.00E-05	0.00395438	yes
ENSMUSG00000027869	Hsd3b6	3:98805503-98814443	152.168	49.5985	1.6173	5.00E-05	0.00395438	yes
ENSMUSG00000027871	Hsd3b1	3:98852193-98859794	5.16614	2.07114	1.31866	5.00E-05	0.00395438	yes
ENSMUSG00000028369	Svep1	4:58042441-58206859	2.23796	1.40039	0.676349	0.0001	0.0071814	yes
ENSMUSG00000028393	Alad	4:62509168-62519918	88.6924	59.6683	0.571846	0.0001	0.0071814	yes
ENSMUSG00000028644	Ermap	4:119175456-119190011	24.5911	11.3026	1.12149	5.00E-05	0.00395438	yes
ENSMUSG00000028716	Pdzk1ip1	4:115088707-115093899	66.3803	30.0747	1.14221	5.00E-05	0.00395438	yes
ENSMUSG00000028717	Tal1	4:115056425-115071755	23.8203	14.4638	0.719751	5.00E-05	0.00395438	yes
ENSMUSG00000028730	Cfap57	4:118554550-118620777	1.47446	0.570909	1.36886	5.00E-05	0.00395438	yes
ENSMUSG00000028825	Rhd	4:134864535-134896172	15.9714	8.46361	0.916146	5.00E-05	0.00395438	yes
ENSMUSG00000029373	Pf4	5:90772434-90773383	27.5113	50.5015	-0.876302	0.00075	0.0361136	yes
ENSMUSG00000029826	Zc3hav1	6:38305285-38354603	8.30176	5.24541	0.662363	0.0002	0.0125309	yes
ENSMUSG00000029922	Mkrn1	6:39397803-39421294	46.5853	25.5015	0.869289	5.00E-05	0.00395438	yes
ENSMUSG00000030000	Add2	6:86028680-86124409	24.2073	11.133	1.1206	5.00E-05	0.00395438	yes
ENSMUSG00000030134	Rasgef1a	6:118011437-118091546	0.195181	0.49823	-1.35199	0.001	0.0449261	yes
ENSMUSG00000030878	Cdr2	7:120957035-120982312	35.9546	21.4777	0.743333	5.00E-05	0.00395438	yes
ENSMUSG00000031431	Tsc22d3	X:140539527-140600659	29.7478	44.3833	-0.577233	0.0002	0.0125309	yes
ENSMUSG00000031451	Gas6	8:13435188-13494490	35.6966	25.0075	0.51343	0.0001	0.0071814	yes
ENSMUSG00000031762	Mt2	8:94170747-94173568	45.2466	23.4717	0.946886	0.0009	0.0416496	yes
ENSMUSG00000032083	Apoa1	9:46228579-46230466	6.12515	16.7289	-1.44952	5.00E-05	0.00395438	yes
ENSMUSG00000032715	Trib3	2:152337421-152344032	7.78329	3.47428	1.16367	0.00015	0.00997772	yes
ENSMUSG00000032890	Rims3	4:120854815-120896579	0.840875	1.53742	-0.87055	0.0001	0.0071814	yes
ENSMUSG00000033214	Slitrk5	14:111675114-111683134	4.62815	11.0012	-1.24915	5.00E-05	0.00395438	yes
ENSMUSG00000033831	Fgb	3:83040140-83049863	0.327422	1.18989	-1.86161	5.00E-05	0.00395438	yes
ENSMUSG00000034059	Ypel4	2:84734057-84738655	14.941	8.94898	0.739478	0.00025	0.0149904	yes

ENSMUSG00000034248	Slc25a37	14:69241847-69305355	99.7739	50.1535	0.992311	5.00E-05	0.00395438	yes
ENSMUSG00000034648	Lrrn1	6:107529767-107570214	3.4459	2.08849	0.722423	0.00095	0.0433331	yes
ENSMUSG00000035551	Igfbpl1	4:45809467-45826923	8.50226	20.9434	-1.30058	5.00E-05	0.00395438	yes
ENSMUSG00000035557	Krt17	11:100256216-100261029	1.90998	0.672897	1.5051	5.00E-05	0.00395438	yes
ENSMUSG00000036198	Arhgap36	X:49463944-49500244	2.95229	0.971798	1.60311	5.00E-05	0.00395438	yes
ENSMUSG00000036306	Lzts1	8:69132668-69184225	0.789793	7.89841	-3.32202	5.00E-05	0.00395438	yes
ENSMUSG00000036390	Gadd45a	6:67035095-67080654	18.871	9.26594	1.02616	0.00015	0.00997772	yes
ENSMUSG00000036832	Lpar3	3:146220962-146286186	15.9628	23.3131	-0.546426	0.00015	0.00997772	yes
ENSMUSG00000037124	Trim58	11:58640464-58652404	1.77528	0.755652	1.23226	0.0002	0.0125309	yes
ENSMUSG00000037254	Itih2	2:10094592-10131396	1.40289	3.33738	-1.25032	5.00E-05	0.00395438	yes
ENSMUSG00000037415	Ranbp10	8:105768307-105827350	13.1638	9.34503	0.494309	0.00045	0.0242455	yes
ENSMUSG00000038402	Foxf2	13:31625815-31631403	0.972717	0.1716	2.50297	5.00E-05	0.00395438	yes
ENSMUSG00000038418	Egr1	18:34859822-34864984	18.2542	12.1865	0.582953	0.0011	0.0483395	yes
ENSMUSG00000038871	Bpgm	6:34476206-34505613	146.542	65.5602	1.16043	5.00E-05	0.00395438	yes
ENSMUSG00000038893	Fam117a	11:95336010-95384507	26.5748	13.7706	0.948465	0.00025	0.0149904	yes
ENSMUSG00000040466	Blvrb	7:27447977-27466144	107.957	67.3931	0.679785	5.00E-05	0.00395438	yes
ENSMUSG00000040713	Creg1	1:165763745-165775308	115.358	68.48	0.752366	0.0007	0.0342417	yes
ENSMUSG00000042045	Sln	9:53850163-53854560	7.88101	4.29752	0.874875	0.0003	0.0174098	yes
ENSMUSG00000042066	Tmcc2	1:132356314-132391281	91.4042	40.8468	1.16204	5.00E-05	0.00395438	yes
ENSMUSG00000042351	Grap2	15:80623504-80650559	12.5722	5.75261	1.12795	5.00E-05	0.00395438	yes
ENSMUSG00000042828	Trim72	7:128001898-128011033	6.29655	3.74696	0.748841	0.00055	0.0283209	yes
ENSMUSG00000044337	Ackr3	1:90203979-90216751	45.9334	32.5207	0.498183	0.0008	0.0378933	yes
ENSMUSG00000044468	Fam46c	3:100451627-100489324	26.6449	9.43641	1.49755	5.00E-05	0.00395438	yes
ENSMUSG00000045287	Rtn4rl1	11:75193782-75267769	6.98447	10.9959	-0.654741	5.00E-05	0.00395438	yes
ENSMUSG00000045394	Epcam	17:87635978-87651106	2.77515	1.07288	1.37108	0.0002	0.0125309	yes
ENSMUSG00000045441	Gprin3	6:59347225-59426294	1.41656	2.20243	-0.636703	0.00045	0.0242455	yes
ENSMUSG00000045545	Krt14	11:100203161-100207548	7.1304	1.28364	2.47375	5.00E-05	0.00395438	yes
ENSMUSG00000045730	Adrb2	18:62177816-62179959	3.91618	1.74278	1.16805	5.00E-05	0.00395438	yes
ENSMUSG00000045763	Basp1	15:25363284-25413764	53.9436	37.6737	0.517892	0.0001	0.0071814	yes
ENSMUSG00000045871	Slitrk6	14:110748577-110755149	1.3606	0.697003	0.965005	0.00095	0.0433331	yes
ENSMUSG00000046182	Gsg1l	7:125878419-126082411	5.54631	8.18374	-0.561232	0.0004	0.0219356	yes
ENSMUSG00000046352	Gjb2	14:57098599-57104702	6.13292	1.98971	1.62401	5.00E-05	0.00395438	yes
ENSMUSG00000048070	Pirt	11:66905720-66947086	6.26579	9.79691	-0.64483	5.00E-05	0.00395438	yes

ENSMUSG00000049409	Prokr1	6:87578590-87590743	3.05108	1.31427	1.21506	0.00015	0.00997772	yes
ENSMUSG00000051839	Gypa	8:80493780-80510542	28.2535	14.9588	0.917436	5.00E-05	0.00395438	yes
ENSMUSG00000051920	Rspo2	15:43020810-43170818	1.09647	0.469546	1.22352	0.0006	0.0304166	yes
ENSMUSG00000052217	Hbb-bh1	7:103841636-103843164	3761.35	1687.86	1.15605	0.00025	0.0149904	yes
ENSMUSG00000052305	Hbb-bs	7:103826533-103828096	3509.86	1116.87	1.65196	5.00E-05	0.00395438	yes
ENSMUSG00000054146	Krt15	11:100131757-100135928	7.47824	1.49289	2.32459	5.00E-05	0.00395438	yes
ENSMUSG00000054191	Klf1	8:84901927-84905291	18.8331	11.2714	0.740608	0.0001	0.0071814	yes
ENSMUSG00000054459	Vsnl1	12:11325246-11436649	24.9591	16.0752	0.634728	5.00E-05	0.00395438	yes
ENSMUSG00000054932	Afp	5:90490736-90515931	16.0833	45.781	-1.50919	0.0003	0.0174098	yes
ENSMUSG00000055401	Fbxo6	4:148145715-148152140	16.5734	9.96639	0.733729	0.00035	0.0196257	yes
ENSMUSG00000058297	Spock2	10:60106218-60135198	1.42397	2.44165	-0.777934	0.0003	0.0174098	yes
ENSMUSG00000058620	Adra2b	2:127363207-127367221	4.00457	2.11821	0.918805	5.00E-05	0.00395438	yes
ENSMUSG00000058794	Nfe2	15:103248211-103258403	32.7873	15.9569	1.03896	5.00E-05	0.00395438	yes
ENSMUSG00000058914	C1qtnf3	15:10952331-10980150	1.0056	0.3409	1.56063	0.0009	0.0416496	yes
ENSMUSG00000059325	Hopx	5:77086987-77115121	88.1615	131.167	-0.573186	5.00E-05	0.00395438	yes
ENSMUSG00000059481	Plg	17:12378608-12419384	0.405986	1.16188	-1.51696	0.00065	0.0322482	yes
ENSMUSG00000060187	Lrrc10	10:117045340-117046768	22.1405	34.2865	-0.630955	5.00E-05	0.00395438	yes
ENSMUSG00000060807	Serpina6	12:103646629-103657212	5.05983	13.6952	-1.43651	5.00E-05	0.00395438	yes
ENSMUSG00000060882	Kcnd2	6:21215502-21729805	0.814022	1.61029	-0.984179	0.00015	0.00997772	yes
ENSMUSG00000061527	Krt5	15:101707069-101712891	13.2029	2.21267	2.57699	5.00E-05	0.00395438	yes
ENSMUSG00000061723	Tnnt3	7:142460808-142516009	119.131	20.6225	2.53026	5.00E-05	0.00395438	yes
ENSMUSG00000062393	Dgkk	X:6779305-6948363	5.50961	7.63851	-0.47134	0.001	0.0449261	yes
ENSMUSG00000063856	Gpx1	9:108338902-108340343	1304.05	707.008	0.883201	5.00E-05	0.00395438	yes
ENSMUSG00000064179	Tnnt1	7:4504569-4516382	189.833	342.617	-0.851867	5.00E-05	0.00395438	yes
ENSMUSG00000064193	Gm4735	2:80837140-80838442	6.40175	3.12695	1.03371	0.00065	0.0322482	yes
ENSMUSG00000068745	Mybphl	3:108364910-108383741	35.3138	24.9172	0.503091	0.0008	0.0378933	yes
ENSMUSG00000069515	Lyz1	10:117287795-117292868	0.607721	2.67963	-2.14056	5.00E-05	0.00395438	yes
ENSMUSG00000069516	Lyz2	10:117277333-117282274	5.42478	18.4367	-1.76494	5.00E-05	0.00395438	yes
ENSMUSG00000069814	Ccdc92b	11:74619604-74641516	6.14091	2.27407	1.43317	5.00E-05	0.00395438	yes
ENSMUSG00000070803	Cited4	4:120666571-120667820	41.0856	27.3857	0.585214	0.0002	0.0125309	yes
ENSMUSG00000071068	Treml2	17:48300037-48312534	2.45959	1.26123	0.96358	0.0001	0.0071814	yes
ENSMUSG00000071604	Fam189a2	19:23972750-24031019	8.66074	13.4005	-0.629727	5.00E-05	0.00395438	yes
ENSMUSG00000071715	Ncf4	15:78244800-78262580	25.5827	9.8877	1.37146	5.00E-05	0.00395438	yes

ENSMUSG00000073063	Hbq1b	11:32286964-32287784	9.07453	3.54481	1.35612	0.00025	0.0149904	yes
ENSMUSG00000073400	Trim10	17:36869573-36877833	27.4735	11.1214	1.3047	5.00E-05	0.00395438	yes
ENSMUSG00000073940	Hbb-bt	7:103812523-103813996	306.665	92.6762	1.72639	5.00E-05	0.00395438	yes
ENSMUSG00000074575	Kcng1	2:168260116-168281736	0.448921	1.27522	-1.50621	0.0005	0.0263692	yes
ENSMUSG00000078137	Ankrd63	2:118699102-118703963	0.282162	1.11627	-1.98409	5.00E-05	0.00395438	yes
ENSMUSG00000078853	Igtp	11:58199555-58222782	6.12169	3.34942	0.870018	0.0009	0.0416496	yes
ENSMUSG00000082101	Slfn14	11:83275109-83286726	6.18061	2.65499	1.21904	5.00E-05	0.00395438	yes
ENSMUSG00000083457	Cyp4b1-ps2	4:115582285-115583144	12.0769	4.50972	1.42114	0.0011	0.0483395	yes
ENSMUSG00000084289	Gm6977	X:90744545-90745062	539.318	284.331	0.923563	5.00E-05	0.00395438	yes
ENSMUSG00000084893	Hba-ps4	17:26286362-26287061	13.7437	4.25302	1.69222	5.00E-05	0.00395438	yes
ENSMUSG00000085700	Hbb-bh0	7:103850019-103850148	4782.05	1515.31	1.65801	5.00E-05	0.00395438	yes
ENSMUSG00000090877	Hspa1b	17:34956435-34959238	2.07742	0.51139	2.0223	5.00E-05	0.00395438	yes
ENSMUSG00000091971	Hspa1a	17:34969189-34972156	2.07014	0.56164	1.88201	5.00E-05	0.00395438	yes
ENSMUSG00000109706	Fth-ps3	8:86902452-86919422	42.0419	22.2895	0.915462	0.00075	0.0361136	yes

Supplemental Table 3: mRNA expression of several transcription factors using RNAseq.

Gene	WW hypoxia (n=3) vs. WW normoxia (n=4) log2(fold change)	*P*
**Tbx5**	0.0264095	0.87225
**Gata4**	0.060515	0.73615
**Mef2c**	0.15607	0.59115
**Hand1**	0.0551793	0.71175
**Hand2**	0.126806	0.37735

Gene	W/R52G normoxia (n=3) vs. WW normoxia (n=4) log2(fold change)	*P*
**Tbx5**	-0.0515783	0.71945
**Gata4**	0.0942385	0.6236
**Mef2c**	0.10706	0.50425
**Hand1**	0.20216	0.51255
**Hand2**	-0.131135	0.37695

Supplemental Figure 1: Difference between the size of fossa ovalis and the length of flap valve in P1 hearts between three experimental groups and control (wild-normoxia).

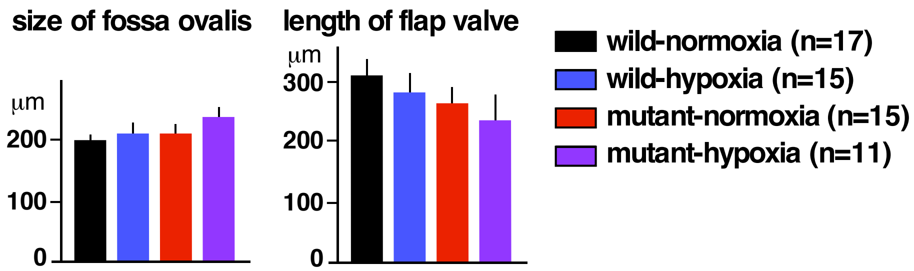

Supplemental Figure 2: Protein expression of several essential cardiac transcription factors under hypoxia relative to normoxic condition. **(A)** Western blotting demonstrating Tbx5, Mef2c, Gata4, Hand1 and GAPDH proteins in wild-normoxia and wild-hypoxia hearts. **(B)** Quantitative data for the protein expression relative to GAPDH. The following antibodies were utilized: Tbx5 (42-6500, Rabbit polyclonal antibody, Thermo Fisher), Mef2c (5030, Rabbit polyclonal antibody, Cell Signaling), Gata4 (AF2606, Goat polyclonal antibody, RD systems), Hand1 (AF3168, Goat polyclonal antibody, RD systems) and GAPDH (MAB374, Mouse monoclonal antibody, Millipore). No significant differences between two groups ($P > 0.05$).

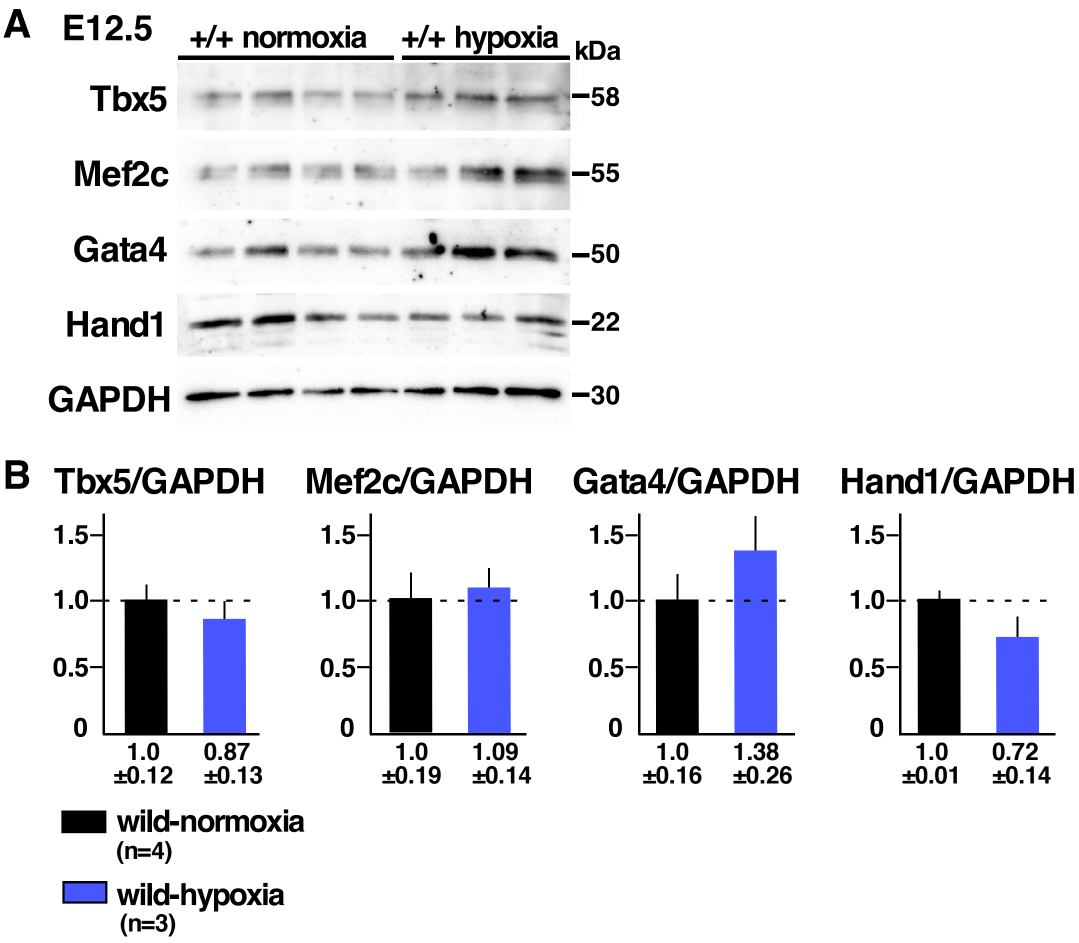

Supplemental Figure 3: Representative images of Nkx2-5 and atrial natriuretic factor (ANF) staining of E12.5 hearts with or without gestational hypoxia in brown color. Enlarged images of Nkx2-5 staining were also shown. ANF expression is higher in left ventricle (LV) compared to right ventricle (RV)(arrows).[1] The following antibodies were utilized: Nkx2-5[2] and ANF (AB5490, Rabbit polyclonal antibody, Millipore).

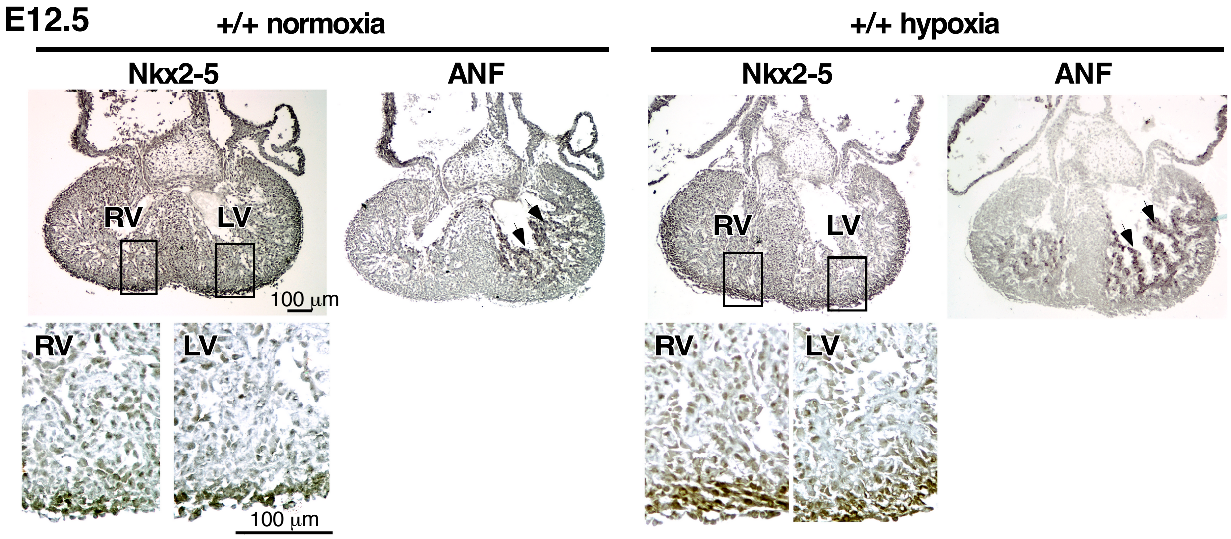

Supplemental Figure 4: Expression of Nkx2-5 mRNA and Hif1α proteins under hypoxia relative to normoxic condition in E18.5 hearts. (**A**) Real-time RT-PCR of Nkx2-5 mRNA relative to ß-actin in E18.5 hearts isolated from wild-normoxia and wild-hypoxia embryos. **(B)** Western blotting demonstrating Hif1α and GAPDH proteins in wild-normoxia and wild-hypoxia hearts. An arrowhead pointed bands migrated around 60 kDa, which are likely degraded form of Hif1α. (**C**) Quantitative data for the 130 kDa Hif1α protein (not degraded) expression relative to GAPDH. The following antibodies were utilized: Hif1α (Ab179483, Rabbit monoclonal antibody, Abcam) and GAPDH (MAB374, Mouse monoclonal antibody, Millipore). No significant differences between two groups ($P > 0.05$).

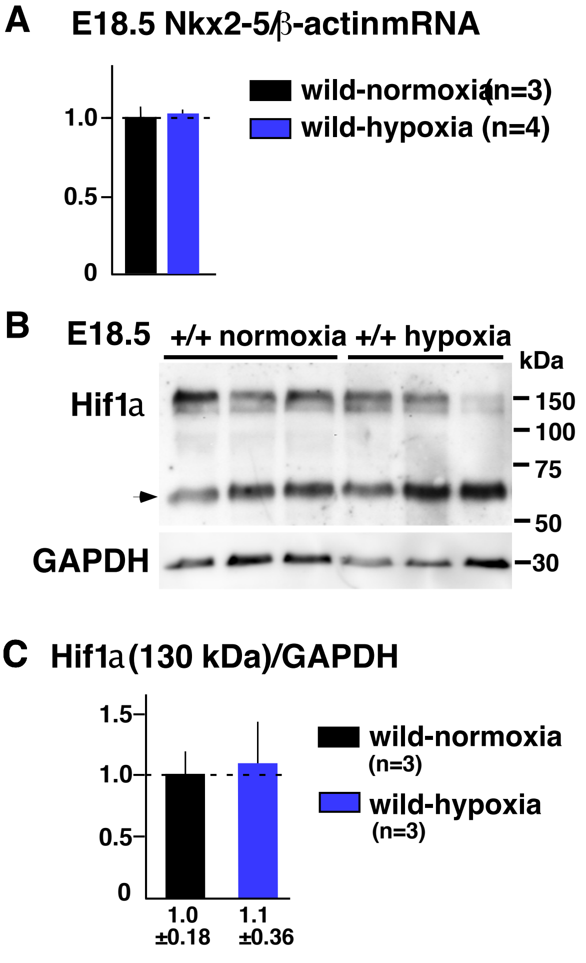

References:

1. Warren SA, Terada R, Briggs LE, Cole-Jeffrey CT, Chien WM, Seki T, Weinberg EO, Yang TP, Chin MT, Bungert J, Kasahara H. Differential role of nkx2-5 in activation of the atrial natriuretic factor gene in the developing versus failing heart. *Mol Cell Biol*. 2011;31:4633-4645.
2. Kasahara H, Bartunkova S, Schinke M, Tanaka M, Izumo S. Cardiac and extracardiac expression of csx/nkx2.5 homeodomain protein. *Circ Res*. 1998;82:936-946.
